# Shedding light on the polyphyletic behavior of the genus *Sterkiella*: The importance of ontogenetic and molecular phylogenetic approaches

**DOI:** 10.1371/journal.pone.0207688

**Published:** 2018-11-20

**Authors:** Daizy Bharti, Santosh Kumar, Govindhasamay R. Varatharajan, Komal Kamra, Antonietta La Terza

**Affiliations:** 1 School of Biosciences and Veterinary Medicine, Laboratory of Animal and Molecular Ecology, University of Camerino, Camerino, MC, Italy; 2 Zoological Survey of India, Prani Vigyan Bhawan, New Alipore, Kolkata, India; 3 Ciliate Biology Laboratory, SGTB Khalsa College, University of Delhi, Delhi, India; Laboratoire de Biologie du Développement de Villefranche-sur-Mer, FRANCE

## Abstract

Present study, investigates a poorly known species of the genus *Sterkiella*, i.e., *S*. *tricirrata*, based on two populations isolated from soil samples collected from the Colfiorito Regional Park, Umbria Region, Italy and from the Silent Valley National Park, India. Both populations showed a highly similar morphology, however different ontogenetic pattern in between. The study confirms the validity of the species *S*. *tricirrata* which was considered to be a species within the *Sterkiella histriomuscorum* complex. The main ontogenetic difference between *S*. *tricirrata* and other species of the genus *Sterkiella* is the different mode of formation of anlagen V and VI of the proter in the former. In the phylogenetic analyses, *Sterkiella tricirrata* clusters with *Sterkiella sinica* within the stylonychine oxytrichids, in a clade away from the type species (*Sterkiella cavicola*) of the genus *Sterkiella*. The study highlights the importance of ontogenetic as well as molecular data in shedding light on the polyphyletic behavior of the genus *Sterkiella*. A detailed description of *S*. *tricirrata* based on morphology, ontogenesis and molecular phylogenetic methods is presented. Further, the improved diagnosis has been provided for the genus *Sterkiella* and the poorly known species *S*. *tricirrata*.

## Introduction

Recent studies, among the hypotrich and spathidiid ciliates, have shown that detailed observations of characters often resolve the discrepancy between the morphological and molecular analyses [1–4]. This reiterates the need for an integrated approach to investigate in-depth ciliate diversity [5]. The identification of cryptic characters among hypotrich ciliates (e.g. cyst structures, morphology, details on the mode of division) has justified the separation of morphologically similar species reflecting distant relationships in molecular phylogeny [3, 6–9]. The structure of resting cyst has provided the support for separation of morphologically similar ciliate species, i.e., cyst species [3, 4, 7–9]. The hypotrich genus *Fragmospina* Foissner, 2016 is a recent example where morphology of cyst has been incorporated as a generic character. Morphologically, *Fragmospina depressa* Foissner, 2016, is rather similar in the arrangement of ciliature with *Sterkiella histriomuscorum* (Foissner et al., 1991) Foissner, Blatterer, Berger & Kohmann, 1991; however, the undulating membranes are arranged in *Australocirrus* pattern [3, 10]. Similarly, when ontogenesis within a genus is compared, most congeners have a rather similar pattern with some minor variations [6]. Kumar et al. [2] erected a new genus, i.e., *Metasterkiella* Kumar et al., 2017, for a species isolated from the petroleum contaminated soil, which was morphologically similar to species of the genus *Sterkiella* Foissner, Blatterer, Berger & Kohmann, 1991. However, this species possesses a variant character in ontogenetic pattern, i.e., it is the only known stylonychid ciliate, thus far, where cirrus V/3 was incorporated during the anlagen formation.

The relatedness between genera within the subfamilies Oxytrichinae and Stylonychinae is rather difficult to understand, despite being strongly reflected as monophyletic groups in the phylogenetic analyses [11–15]. A probable explanation could be the insufficient availability and interpretation of morphological and ontogenetic data for the known species. Previous studies have shown that the stylonychid genus *Sterkiella* is a non-monophyletic assemblage [1, 2, 16, 17]. *Sterkiella histriomuscorum*, a sibling species complex according to Berger [11], include populations which have not been studied in detail and thus considered to be synonyms under the complex.

In this study, we describe a poorly known species *Sterkiella tricirrata* (Buitkamp, 1977) Berger, 1999, which is probably a synonym of one of the species within the *Sterkiella histriomuscorum* complex together with *S*. *terricola*, according to the remarks of Berger [11]. The Indian and Italian populations of *S*. *tricirrata* were studied and found to be highly similar in morphology. Ontogenetic stages of both populations showed difference in the anlagen formation during the early divisional stages with respect to that of *Sterkiella* species [11, 18, 19], and thus suggesting its possible separation at the genus level. Detailed data on the morphometry and ontogenesis for both populations and molecular analyses based on SSU rRNA gene of Italian population is presented. Furthermore, this study highlights the relevance of combining ontogenetic and molecular phylogenetic approaches in identifying species in polyphyletic assemblages such as that represented by the stylonychid genus *Sterkiella*.

## Materials and methods

### Description of the sampling site and sample processing

Soil samples were collected from the core zone of the Silent Valley National Park, India (11°08' 40.72"N; 129°20' 38"E) in January, 2008 and from the plains of the Colfiorito and Plestini uplands, Umbria region, Central Italy (43°01' 40.72"N; 12°52' 39.46"E) in July, 2009. Vegetative cells were excysted from resting cysts from two-weeks-dried soil samples (approximately 200 g) by employing the non-flooded Petri dish method [20]. A clonal culture of *Sterkiella tricirrata* was established for both the populations as described in Kumar et al. [1], i.e., using Pringsheim’s medium for culturing and the green alga *Chlorogonium elongatum* as food source. Observations on the live specimens were made using a microscope with bright-field and differential interference contrast illuminations at a magnification of 100–1000×. The protargol staining method described by Kamra and Sapra [21] was used with some modification to reveal the ciliature. Measurements of impregnated specimens were performed at a magnification of 1000× using an ocular micrometer for Italian population and Leica software IM50 image manager for the Indian population. An Optika microscope camera was employed for photomicrography for the Italian population and Leica camera DFC320 for the Indian. The illustration of the live specimen was prepared using free-hand sketches, while those of impregnated specimens were made with the drawing device. Terminology is according to Berger [11] and Wallengren [22].

### DNA extraction, PCR amplification, and sequencing

Unfortunately, we could not perform the DNA extraction for the Indian population. Thus the methods described here, including the phylogenetic analyses of the SSU rRNA gene, exclusively refer to the Italian population. Five cells were collected from a clonal culture with the help of glass micropipettes and washed three times with autoclaved distilled water (same culture was used for live observation and protargol staining to study morphology and ontogenesis). Genomic DNA was extracted using the Norgen DNA Kit (Elettrofor Scientific Instruments, Borsea, Italy), following the manufacturer’s instruction [23]. Extracted DNA (5 μl) was dispensed into a PCR tube containing 5 μl of autoclaved distilled water, and amplifications were carried out using high-fidelity Pfx50 DNA polymerase (Invitrogen, Italy) in a total volume of 50 μl with the universal eukaryotic primers Euk A (FW 5’-AACCTGGTTGATCCTGCCAGT-3’) and Euk B (RV 5’-TGATCCTTCTGCAGGTTCACCTAC- 30) [24]. Additionally, nested primer pairs Eup 18S (FW 5’-TAG AGG GAC TTT GTG TGC AAC C-3’) and Eup 18S (RV 5’-ATC TCC CTG AAA CAC ACG TTG G-3’) were used in combination with the universal primers for amplification and sequencing. The PCR program for 18S rDNA amplification included an initial denaturation at 94°C for 3 min, followed by 35 cycles of 94°C for 1 min, 55°C for 45 s and 72°C for 80 s, with a final extension step at 72°C for 10 min. After confirmation of the appropriate size, the PCR products were purified using the Nucleospin gel extraction kit (Qiagen) and were then directly sequenced on both strands at StarSEQ GMBH, Germany.

### Phylogenetic analyses

For phylogenetic analyses, the SSU rRNA gene sequence of *Sterkiella tricirrata* was aligned with 55 SSU rRNA gene sequences of hypotrich ciliates from GenBank using the MAFFT software v. 7.047 (choosing the iterative refinement methods Q-INS-I that considers the secondary structure of the SSU rRNA molecules) [25].

Ambiguously aligned regions were identified and excluded from the phylogenetic analyses with GBlocks v.0.91b [26] using parameters optimized for rRNA alignments (minimum length of A block = 5, allowed gap positions = with half), leaving 1,644 unambiguous positions. The final alignment was then used for subsequent phylogenetic analyses after converting the FASTA (.fas) file to NEXUS (.nex) format using the open web-based tool ALTER (Alignment Transformation EnviRonment) [27]. A Bayesian inference (BI) analysis was performed using MrBayes v.3.2.1 [28] and the GTR+I+G model, as selected by the jModel Test v.2.1.3 software [29] under the *Akaike Information Criterion corrected* (AICc). Markov chain Monte Carlo (MCMC) simulations were run, with two sets of four chains using the default settings, for 1,000,000 generations with trees sampled every 100 generations and discarding the first 25% of the sampled trees as burn-in. The remaining trees were used to generate a consensus tree and to calculate the posterior probabilities (PP) of all branches using the majority-rule consensus approach. The previous alignment was also used to perform a Maximum Likelihood (ML) tree by means of the Molecular Evolutionary Genetic Analysis (MEGA) software, v.5.2.2 [30] using the default parameters and the GTR+I+G model. The reliability of tree topology was assessed by 1,000 bootstrap replicates and was expressed as a percentage. Phylogenetic trees were visualized using the free software package FigTree v1.4 by A. Rambaut at http://tree.bio.ed.ac.uk/software/figtree/.

### Data availability

The newly obtained SSU rRNA gene sequence of *Sterkiella tricirrata* is available from the GenBank/EMBL databases (accession number: MG805314). Two neotype slides of the Italian population containing the protargol stained neotype specimen and relevant morphostatic specimens have been deposited at the Natural History Museum, London, UK, with registration numbers NHMUK 2014.3.20.1 and NHMUK 2014.3.20.2. Further, two slides of the Indian population is deposited at the Natural History Museum, London, UK, with registration numbers NHMUK 2011.7.4.2 and NHMUK 2011.7.4.3 and one at the type collection of the Zoological Survey of India, Kolkata, India, with registration number Pt 3067. The SSU rRNA gene sequence is deposited in GenBank (accession number: MG805314).

### Nomenclatural acts

The electronic edition of this article conforms to the requirements of the amended International Code of Zoological Nomenclature, and hence the new names contained herein are available under that Code from the electronic edition of this article. This published work and the nomenclatural acts it contains have been registered in ZooBank, the online registration system for the ICZN. The ZooBank LSIDs (Life Science Identifiers) can be resolved and the associated information viewed through any standard web browser by appending the LSID to the prefix “http://zoobank.org/. The LSID for this publication is: urn:lsid:zoobank.org:pub:DB29FEE1-22B6-48CC-9E8D-661AD15BBB06. The electronic edition of this work was published in a journal with an ISSN, and has been archived and is available from the following digital repositories: PubMed Central, LOCKSS.

## Results

### Description of *Sterkiella tricirrata*

**Fig 1:**
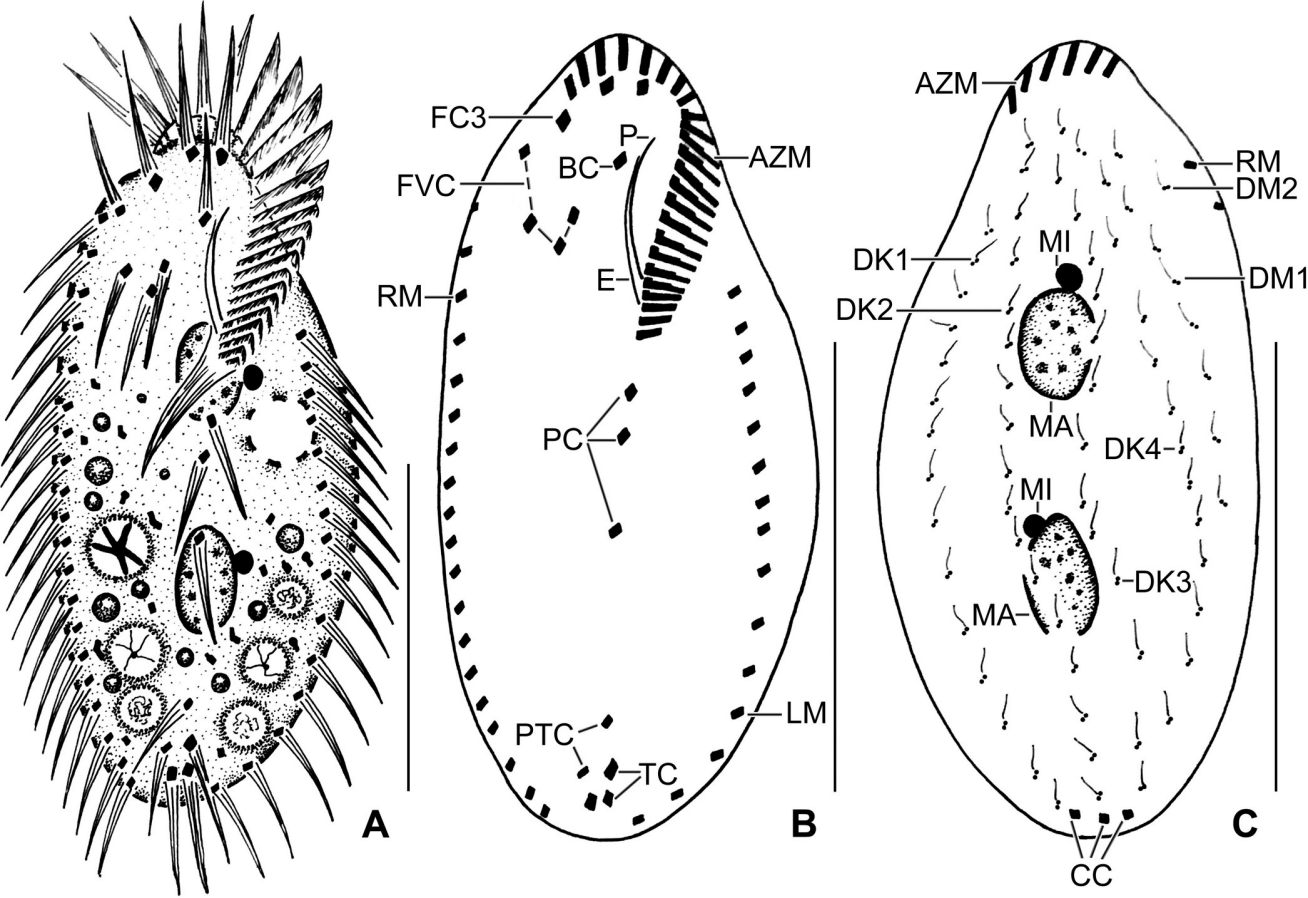
**Line diagrams of *Sterkiella tricirrata* Italian population from life (A) and after protargol impregnation (B, C).** **(A)** A representative cell with a length of 85 μm. **(B, C)** Ventral and dorsal views of a voucher specimen, showing the ciliature and the nuclear apparatus. Note the invariably three transverse cirri typical of the species. AZM, adoral zone of membranelles; BC, buccal cirrus; CC, caudal cirri; DK1–4, dorsal kineties; DM1,2, dorsomarginal kineties; E, endoral membrane; FC3, frontal cirrus 3; FVC, frontoventral cirri; LM, left marginal row; MA, macronuclear nodules; MI, micronuclei; P, paroral membrane; PC, postoral cirri; PTC< pretransverse cirri; RM, right marginal row. Scale bars = 40 μm.

**Fig 2:**
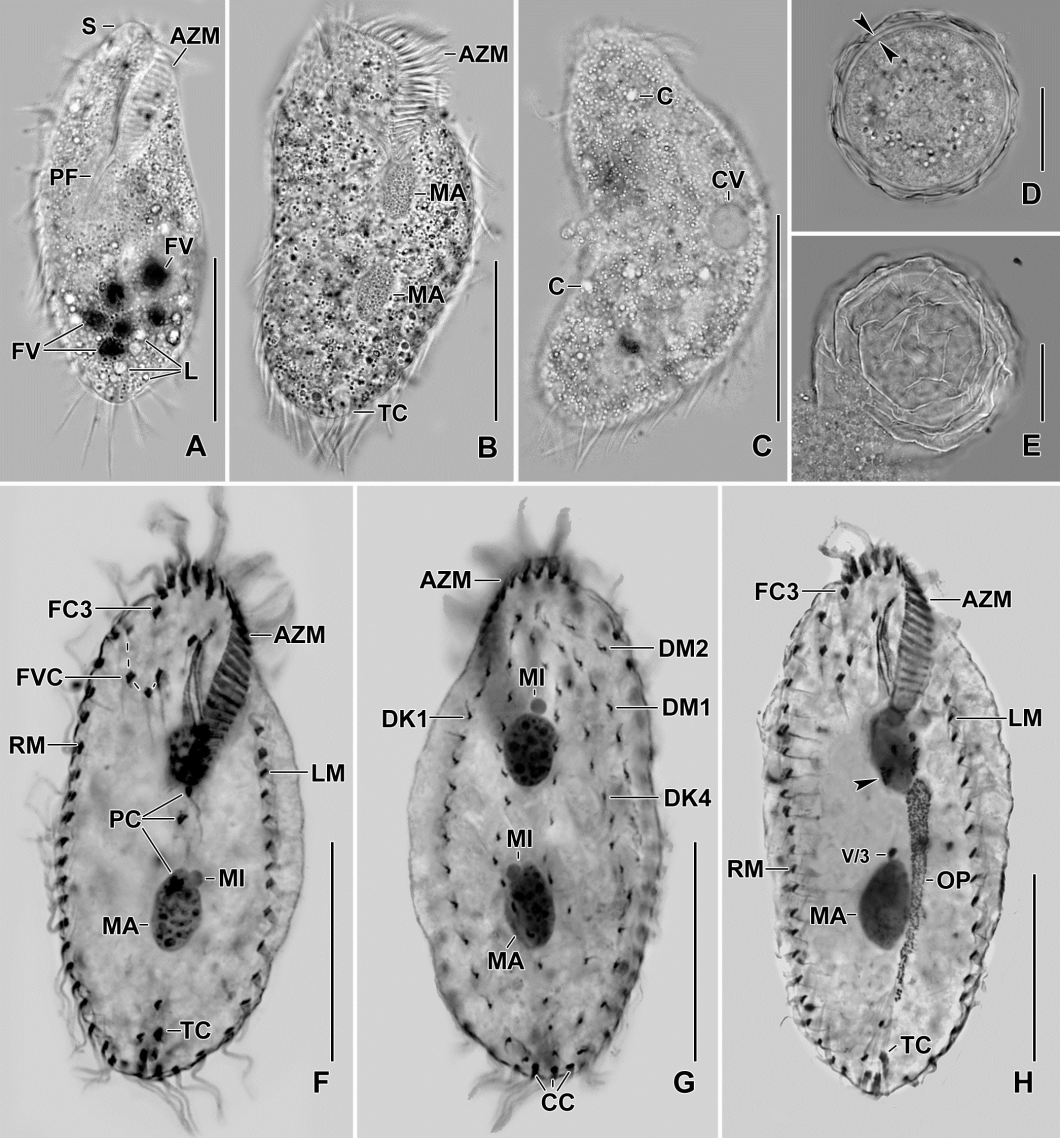
**Photomicrographs of *Sterkiella tricirrata* Italian population from life (A–E) and after protargol impregnation (F–H).** (**A)** Specimen, showing body shape, food vacuoles and lipid droplets. **(B, C)** Slightly compressed specimens due to cover slip pressure, showing nuclear apparatus (B), cytoplasmic crystals and contractile vacuole (C). **(D, E)** Resting cyst. Optical section (D), showing the cyst wall (opposed arrowheads). Squeezed cyst (E) with contents released, showing the wrinkled hyaline ridges. **(F, G)** Ventral view of the main voucher specimen, showing body shape, nuclear apparatus, and ciliature of the ventral (F) and dorsal surface (G). **(H)** An early divider, showing the formation of oral primordium close to transverse cirri. AZM, adoral zone of membranelles; C, crystals; CC, caudal cirri; CV, contractile vacuole; DK1,4, dorsal kineties; DM1,2, dorsomarginal kineties; FC3, frontal cirrus 3; FV, food vacuoles; FVC, frontoventral cirri; L, lipid droplets; LM, left marginal row; MA, macronuclear nodules; MI, micronuclei; OP, oral primordium; PC, postoral cirri; PF, pharyngeal fibre; RM, right marginal row; S, scutum; TC, transverse cirri; V/3, postoral ventral cirrus. Scale bars = 15 μm (D, E) and 30 μm (A–C, F–H).

**Fig 3:**
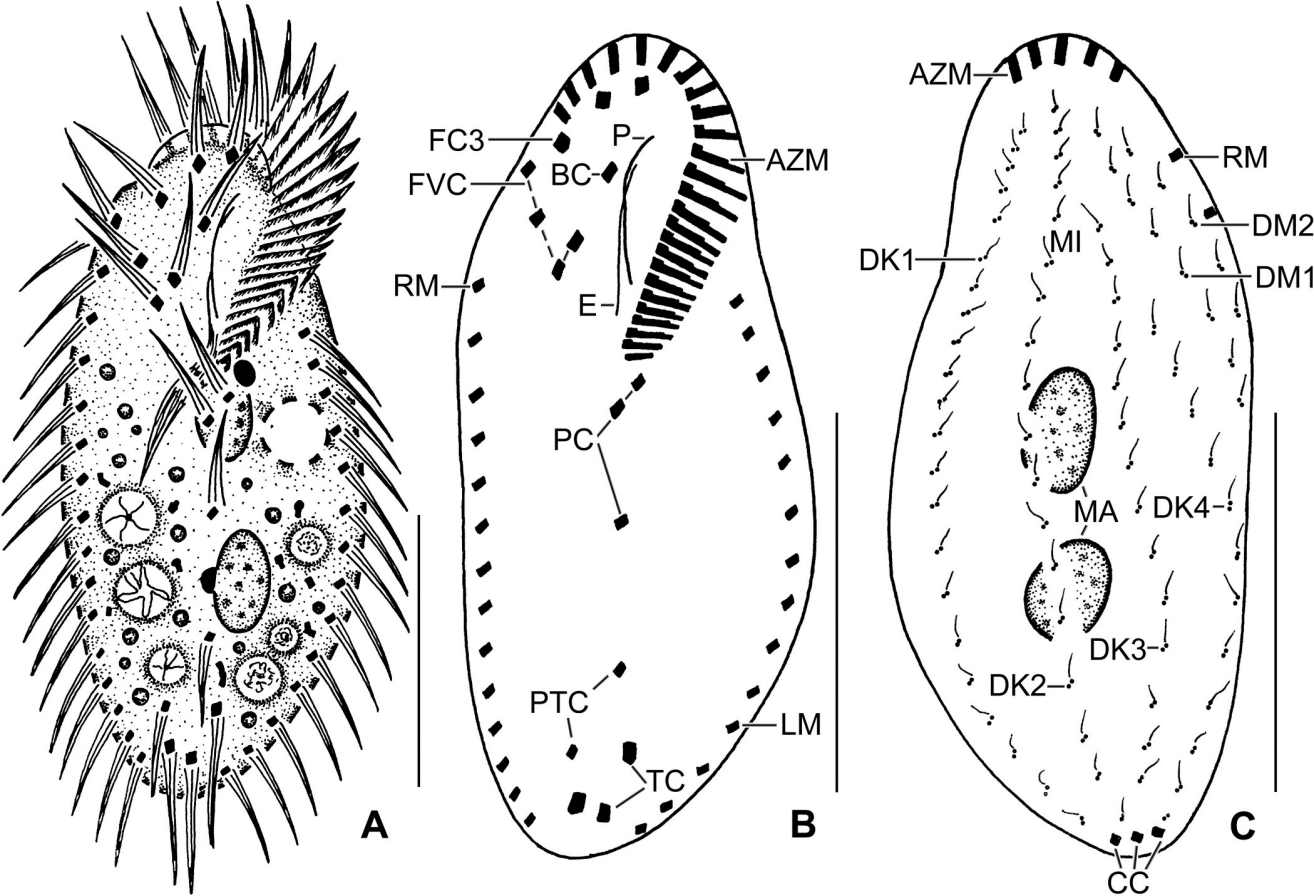
**Line diagrams of *Sterkiella tricirrata* Indian population from life (A) and after protargol impregnation (B, C).** **(A)** A representative cell with a length of 75 μm. **(B, C)** Ventral and dorsal views of a voucher specimen, showing the ciliature and the nuclear apparatus. Note the invariably three transverse cirri. AZM, adoral zone of membranelles; BC, buccal cirrus; CC, caudal cirri; DK1–4, dorsal kineties; DM1,2, dorsomarginal kineties; FC3, frontal cirri; FVC, frontoventral cirri; LM, left marginal row; MA, macronuclear nodules; P, paroral membrane; PC, postoral cirri; PTC, pretransverse cirri; RM, right marginal row; TC, transverse cirri. Scale bars = 30 μm.

**Fig 4:**
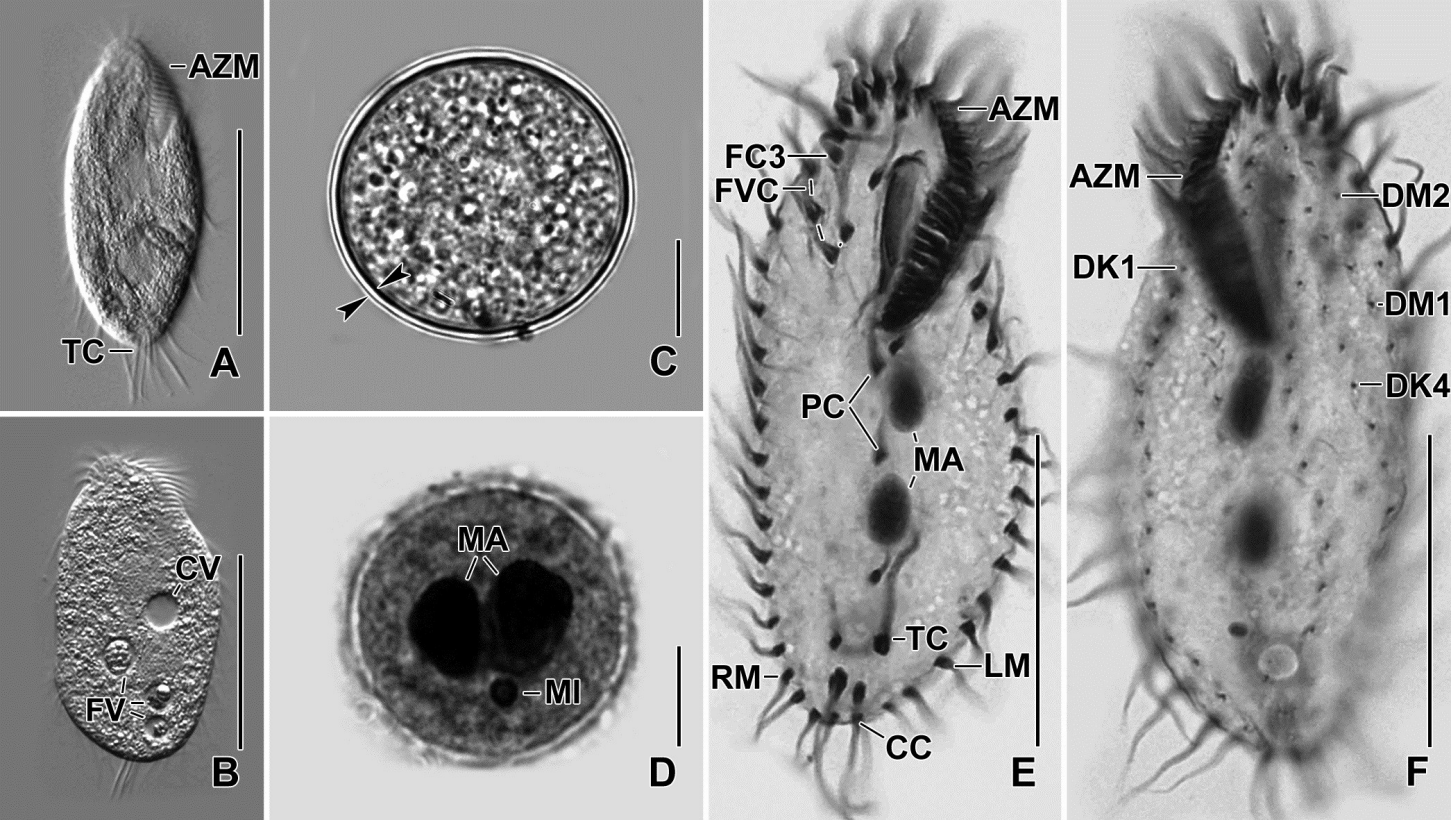
**Photomicrographs of *Sterkiella tricirrata* Indian population from life (A–C) and after protargol impregnation (D–F).** (**A)** Slightly starved specimen, showing body shape and three transverse cirri protruding beyond posterior end of the cell. **(B)** Slightly compressed specimen due to cover slip pressure, showing food and contractile vacuole. **(C, D)** Resting cyst. Optical section (D), opposed arrowheads mark the cyst wall (C). Macronuclear nodules are separate in mature cyst (D). **(E, F)** Ventral view of the specimen, showing body shape, nuclear apparatus, and ciliature of the ventral (E) and dorsal surface (F). AZM, adoral zone of membranelles; CC, caudal cirri; CV, contractile vacuole; DK1,4, dorsal kineties; DM1,2, dorsomarginal kineties; FC3, frontal cirri; FV, food vacuole; FVC, frontoventral cirri; LM, left marginal row; MA, macronuclear nodules; MI, micronuclei; PC, postoral cirri; RM, right marginal row; TC, transverse cirri. Scale bars = 10 μm (C, D), 30 μm (E, F) and 40 μm (A, B).

Morphometric data of the Indian and Italian population of *Sterkiella tricirrata* highly overlap (Table 1). Thus only a detailed description of the Italian population is provided below; minor differences with the Indian population in some characters include: (1) body size, i.e., about 85 × 40 μm (vs. 75 × 40 μm) in vivo; (2) number of cirri in right marginal rows 20 (vs. 16); and (3) number of bristles in first dorsal kinety (15 vs. 20) (Figs 1A–1C, 2A–2C, 2F, 2G, 3A–3C, 4A, 4B, 4E and 4F and Table 1).

**Table 1:** Morphometric data on Italian (ITA) and Indian (IND) populations of *Sterkiella tricirrata*.

Characteristic^a^	Population	Mean	M	SD	SE	CV	Min	Max	n
Body, length	ITA	75.3	75.0	6.6	1.4	8.8	63.0	86.0	21
	IND	65.7	65.2	3.9	1.0	5.9	60.8	74.2	15
Body, width	ITA	34.3	35.0	4.3	0.9	12.4	26.0	40.0	21
	IND	30.4	29.3	3.2	0.8	10.4	26.1	38.2	15
Body length:width, ratio	ITA	2.2	2.2	0.2	0.0	9.0	1.9	2.6	21
	IND	2.2	2.2	0.2	0.0	7.7	1.9	2.5	15
Anterior body end to proximal end of adoral zone, distance	ITA	26.4	27.0	1.6	0.3	5.9	22.0	29.0	21
	IND	25.2	25.4	1.6	0.4	6.4	22.6	28.3	15
Body length:AZM length, ratio	ITA	2.9	2.9	0.2	0.0	6.7	2.5	3.2	21
	IND	2.6	2.6	0.1	0.0	5.5	2.5	3.1	15
Anterior body end to proximal end of adoral zone, % of body length	ITA	35.2	34.7	2.4	0.5	6.9	31.4	40.6	21
	IND	38.4	38.4	1.9	0.5	5.0	32.8	40.9	15
DE-value^b^	ITA	0.2	0.2	0.0	0.0	19.9	0.1	0.3	15
	IND	0.3	0.3	0.0	0.0	17.1	0.2	0.3	11
Adoral membranelles, number	ITA	22.9	23.0	1.3	0.3	5.8	20.0	26.0	21
	IND	24.0	24.0	1.4	0.4	5.7	21.0	26.0	15
Adoral membranelles, width of largest base	ITA	5.4	5.0	0.5	0.1	8.9	5.0	6.0	21
	IND	5.8	6.0	0.6	0.2	10.4	5.0	7.0	11
Anterior body end to paroral membrane, distance	ITA	7.9	8.0	0.8	0.2	10.6	6.5	9.0	21
	IND	7.5	8.0	1.1	0.3	15.1	5.0	9.0	11
Anterior body end to anterior macronuclear nodule, distance	ITA	19.2	20.0	2.6	0.6	13.3	15.0	24.0	21
	IND	24.4	24.0	2.6	0.8	10.6	19.0	29.0	11
Anterior macronuclear nodule, length	ITA	10.9	11.0	1.6	0.4	14.8	8.0	13.0	21
	IND	8.8	8.4	1.3	0.3	14.9	7.0	12.2	15
Anterior macronuclear nodule, width	ITA	7.2	7.0	0.9	0.2	12.1	5.0	9.0	21
	IND	5.3	5.4	0.4	0.1	8.3	4.7	6.3	15
Posterior macronuclear nodule, length	ITA	11.7	11.0	2.3	0.5	19.6	8.0	17.0	21
	IND	8.6	9.0	1.2	0.4	14.0	7.0	11.0	11
Posterior macronuclear nodule, width	ITA	6.8	7.0	1.0	0.2	15.1	5.0	9.0	21
	IND	5.7	6.0	0.8	0.2	13.7	5.0	7.0	11
Macronuclear nodules, number	ITA	2.0	2.0	0.0	0.0	0.0	2.0	2.0	21
	IND	2.0	2.0	0.0	0.0	0.0	2.0	2.0	21
Anterior micronucleus, diameter	ITA	2.2	2.2	0.2	0.0	10.1	1.8	2.5	21
	IND	1.9	1.8	0.1	0.0	7.0	1.7	2.1	15
Micronuclei, number	ITA	2.0	2.0	0.7	0.1	34.3	1.0	4.0	21
	IND	2.0	2.0	0.0	0.0	0.0	2.0	2.0	15
Anterior body end to right marginal row, distance	ITA	14.1	14.0	1.5	0.4	10.7	10.0	16	15
	IND	16.0	16.0	2.1	0.6	13.4	12.0	19.0	11
Posterior body end to right marginal row, distance	ITA	5.3	5.0	1.3	0.3	24.7	4.0	8.0	15
	IND	3.6	4.0	1.1	0.3	30.8	2.0	5.0	11
Right marginal row, number of cirri	ITA	19.7	20.0	1.1	0.2	5.6	18.0	21.0	21
	IND	15.7	16.0	0.9	0.2	5.7	14.0	18.0	15
Anterior body end to left marginal row, distance	ITA	22.5	23.0	1.6	0.4	6.9	20.0	25.0	15
	IND	21.3	21.0	1.4	0.4	6.7	20.0	24.0	11
Posterior body end to left marginal row, distance	ITA	1.5	1.0	0.8	0.2	53.0	1.0	3.0	15
	IND	2.2	2.0	0.8	0.2	34.4	1.0	3.0	11
Left marginal row, number of cirri	ITA	16.4	16.0	1.2	0.3	7.3	14.0	18.0	21
	IND	14.3	14.0	1.0	0.2	6.7	13.0	16.0	15
Gap between last cirri of marginal rows	ITA	9.9	10.0	1.4	0.4	14.5	7.0	12.0	15
Frontal cirri, number	ITA	3.0	3.0	0.0	0.0	0.0	3.0	3.0	21
	IND	3.0	3.0	0.0	0.0	0.0	3.0	3.0	21
Anterior body end to buccal cirrus, distance	ITA	9.7	10.0	0.9	0.2	9.4	8.0	11.0	21
	IND	10.7	11.0	1.3	0.4	11.9	8.0	12.0	11
Anterior of paroral to buccal cirrus, distance	ITA	1.9	2.0	0.5	0.1	26.5	1.0	3.0	21
	IND	3.5	3.0	0.5	0.2	15.1	3.0	4.0	11
Buccal cirrus, number	ITA	1.0	1.0	0.0	0.0	0.0	1.0	1.0	21
	IND	1.0	1.0	0.0	0.0	0.0	1.0	1.0	21
Frontoventral cirri, number	ITA	4.0	4.0	0.0	0.0	0.0	4.0	4.0	21
	IND	4.0	4.0	0.0	0.0	0.0	4.0	4.0	21
Distance between cirrus V/2 and V/3	ITA	19.3	19.0	4.1	1.0	21.1	10.0	27.0	15
	IND	10.6	10.0	1.9	0.6	17.4	9.0	15.0	11
Postoral cirri, number	ITA	3.0	3.0	0.0	0.0	0.0	3.0	3.0	21
	IND	3.0	3.0	0.0	0.0	0.0	3.0	3.0	21
Pretransverse cirri, number	ITA	2.0	2.0	0.0	0.0	0.0	2.0	2.0	21
	IND	2.0	2.0	0.0	0.0	0.0	2.0	2.0	21
Posterior body end to rear transverse cirrus, distance	ITA	3.1	3.0	0.9	0.2	29.2	1.0	4.0	15
	IND	3.5	3.0	0.7	0.2	19.9	3.0	5.0	11
Transverse cirri, number	ITA	3.0	3.0	0.0	0.0	0.0	3.0	3.0	21
	IND	3.0	3.0	0.0	0.0	0.0	3.0	3.0	21
Dorsal kineties, number	ITA	6.0	6.0	0.0	0.0	0.0	6.0	6.0	21
	IND	6.0	6.0	0.0	0.0	0.0	6.0	6.0	15
Anterior body end to dorsal kinety 1, distance	ITA	16.4	17.0	2.1	0.6	13.0	11.0	19.0	15
Dorsal kinety 1, number of bristles	ITA	14.6	15.0	1.3	0.3	8.8	13.0	18.0	21
	IND	19.9	20.0	1.7	0.4	8.7	17.0	22.0	15
Dorsal kinety 2, number of bristles	ITA	16.4	16.0	1.7	0.4	10.7	14.0	21.0	21
	IND	17.0	17.0	1.7	0.4	10.2	14.0	20.0	15
Dorsal kinety 3, number of bristles	ITA	11.3	11.0	1.1	0.3	10.2	10.0	14.0	21
	IND	11.7	12.0	2.1	0.5	17.5	7.0	14.0	15
Dorsal kinety 4, number of bristles	ITA	9.9	10.0	1.0	0.2	10.0	8.0	13.0	21
	IND	12.4	12.0	1.4	0.3	10.9	11.0	15.0	15
Dorsomarginal row 1, number of bristles	ITA	6.9	7.0	0.9	0.2	12.4	5.0	8.0	21
	IND	8.3	8.0	0.8	0.2	9.8	7.0	10.0	15
Dorsomarginal row 2, number of bristles	ITA	2.9	3.0	0.8	0.2	26.5	2.0	5.0	21
	IND	4.8	5.0	0.8	0.2	16.1	3.0	6.0	15
Caudal cirri, distance in between	ITA	5.7	5.5	0.7	0.2	11.9	5.0	7.0	15
Caudal cirri, number	ITA	3.1	3.0	0.3	0.1	9.7	3.0	4.0	21
	IND	3.0	3.0	0.0	0.0	0.0	3.0	3.0	15

a Data based on mounted, protargol-impregnated, and randomly selected specimens from the clonal cultures of Italian and Indian populations fed with *Chlorogonium elongatum*. Measurements in μm. CV–coefficient of variation in %, M–median, Max–maximum, Mean–arithmetic mean, Min–minimum, n–number of individuals investigated, SD–standard deviation, SE–standard error of arithmetic mean.

b *Distal End* of adoral zone [12]

Size in vivo 70–90 × 30–50 μm, usually about 85 × 40 μm, as calculated from some in vivo (n = 6) measurements and morphometric data in Table 1, assuming 15% preparation shrinkage [9]. Body outline oval, elliptical to broadly elliptical, both ends rounded; dorso-ventrally flattened about 2:1 (Figs 1A–1C, 2A–2C, 2F and 2G and Table 1). Nuclear apparatus in or slightly left of midline composed of two macronuclear nodules and one to four micronuclei (Figs 1A, 1C, 2B, 2F and 2G and Table 1). Macronuclear nodules ellipsoidal to broadly ellipsoidal, anteriormost nodule on average 11 × 7 μm in protargol preparations; contain small nucleoli, 1–3 μm across. Micronuclei usually attached to macronuclear nodules, globular, on average 2.0 μm across in protargol preparations (Figs 1A, 1C, 2B, 2F and 2G and Table 1). Contractile vacuole slightly anterior of body’s midline, near left cell margin (Figs 1A and 2C). Cortex semirigid; cortical granules absent. Cytoplasm colorless, filled with some crystals of about 1–3 μm in size and fat droplets about 2–6 μm in diameter (Figs 1A, 2A and 2B). Feeds on bacteria and small flagellates in non-flooded Petri dish culture (Figs 1A and 2A). Movement by rapid crawling over and between soil particles.

Cirral pattern and number of cirri rather constant. Invariably, 16 fronto-ventral-transverse cirri (Figs 1A, 1B and 2F and Table 1). Three frontal cirri, in vivo about 15 μm long, right cirrus posterior of distal end of adoral zone, middle cirrus anterior of buccal cirrus, left cirrus anterior of distal end of undulating membranes. One buccal cirrus about 10 μm distant from anterior body end in protargol preparation. Four frontoventral cirri, arranged in opposed J-shaped pattern (Figs 1A, 1B and 2F and Table 1). Three postoral cirri behind buccal vertex (distance between cirrus V/3 and V/4 is double to that of cirrus IV/2 and V/4) and two slightly obliquely arranged pretransverse cirri. Invariably, three transverse cirri, in vivo about 14 μm long, base of rearmost cirrus about 3 μm distant from posterior body end (Figs 1A, 1B, 2B and 2F and Table 1). Marginal rows non-confluent posteriorly, cirri about 13 μm long in protargol preparations. Left row composed of an average of 16 cirri; right row about 5 μm distant from posterior body end, composed of an average of 20 cirri (Figs 1A–1C and 2F and Table 1).

Invariably six dorsal kineties with bristles about 2–3 μm long in protargol preparations. Kinety 1 and 4 shortened anteriorly, kineties 2–3 bipolar, kineties 5 and 6 distinctly shortened posteriorly (Figs 1C and 2G and Table 1). Three caudal cirri, one each at posterior ends of dorsal kineties 1, 2, and 4 (Figs 1C and 2G and Table 1).

Adoral zone extends about 35% of body length, on average composed of 23 membranelles with about 15 μm long cilia in vivo, bases of largest membranelles about 5 μm wide in protargol preparations (Figs 1A–1C, 2A, 2B and 2F and Table 1). Undulating membranes left of body’s midline, slightly curved, intersect optically near anterior third or remains parallel. Paroral commences about 8 μm from anterior body end; endoral commences at the level of buccal cirrus (Figs 1A, 1B, 2A, 2B and 2F and Table 1).

### Resting cyst

Resting cysts (two-week-old) about 35 μm across in vivo; cyst surface with hyaline ridges, about 3.0 μm high (Fig 2D and 2E). Cyst wall 1.0–1.5 μm thick. Nuclear apparatus with separate macronuclear nodules (Fig 2D, 4C and 4D). Cyst content includes many lipid droplets 1.5–3.0 μm across in vivo (Fig 2D and 2E).

### Notes on ontogenesis

**Fig 5:**
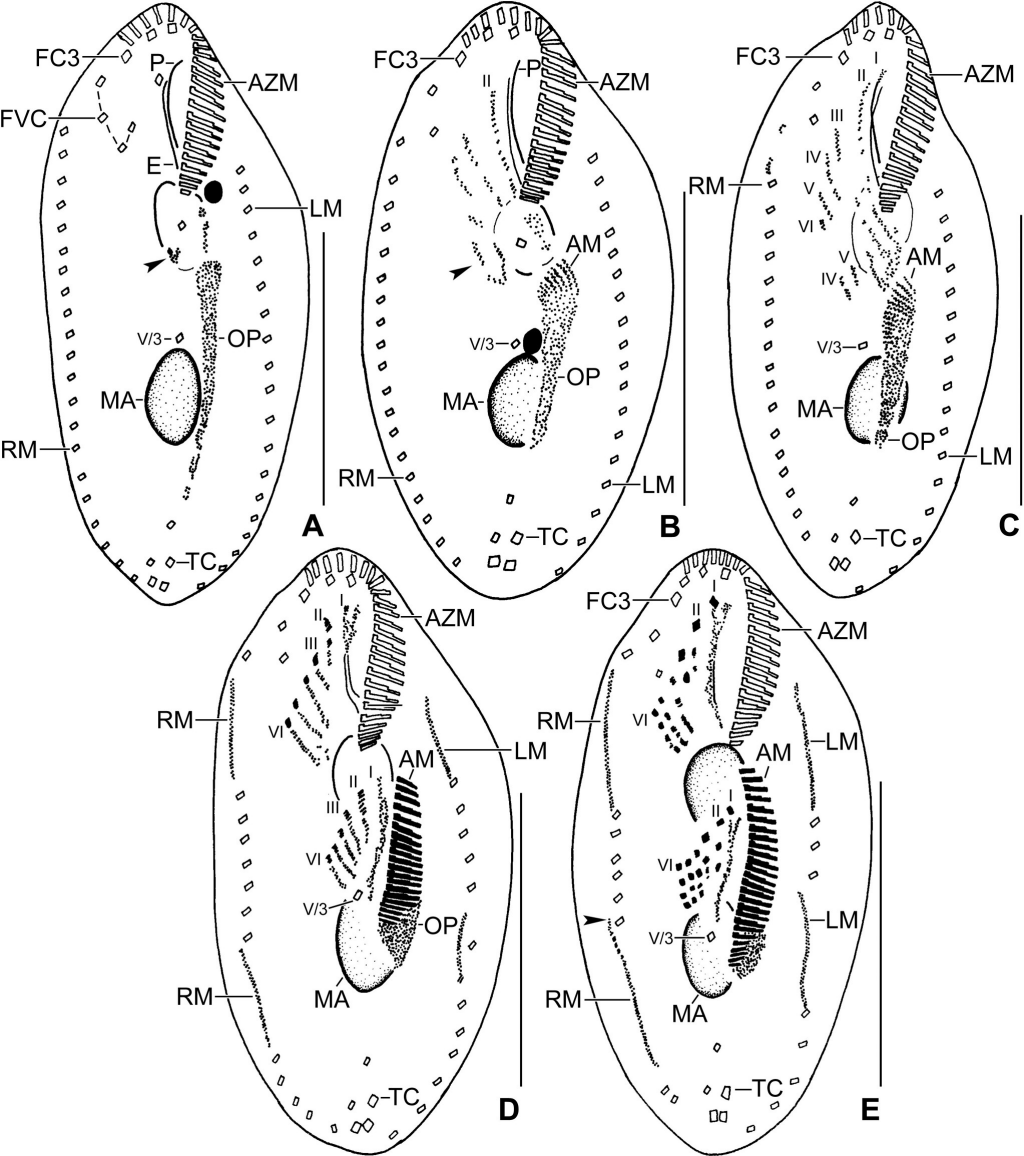
Line diagrams of protargol stained early dividers of *Sterkiella tricirrata* Italian population. **(A)** Arrowheads in (A, B) mark the disaggregating cirrus V/4 forming two anlagen, anterior portion of which proliferates anteriorly forming anlagen V and VI of the proter. **(B)** Two anlagen arise from the anterior end of the oral primordium. Anlage II of the opisthe moves anteriorly, part of this anlage merges with the parental buccal cirrus in early divider. Anlage III moves anteriorly and merges with the cirrus III/2. **(C)** Cirrus IV/3 disaggregates and forms anlage IV of the proter. Cirrus IV/2 forms the anlage IV of the opisthe. **(D)** Six anlagen are formed both for the proter and the opisthe. Posterior ends of the anlagen II to IV of the opisthe separates and form anlage I. Four anlagen for marginal cirri develop incorporating four to five parental marginal cirri in the proter and the opisthe. **(E)** Anlage I of the proter develops by partial reorganization of the paroral and endoral. Overall, five parental cirri (II/2, III/2, IV/3, IV/2, and V/4) disaggregate to give rise to five fronto-ventral–transverse anlagen for the proter and the opisthe. Arrowhead points to the anlagen of the dorsomarginal kineties. AM, adoral membranelles; AZM, adoral zone of membranelles; E, endoral membrane; FC3, frontal cirrus 3; FVC, frontoventral cirri; LM, left marginal row; MA, macronuclear nodules; OP, oral primordium; P, paroral membrane; RM, right marginal row; TC, transverse cirri; V/3, postoral ventral cirrus. Numerals denote cirral anlagen. Scale bars = 40 μm.

**Fig 6:**
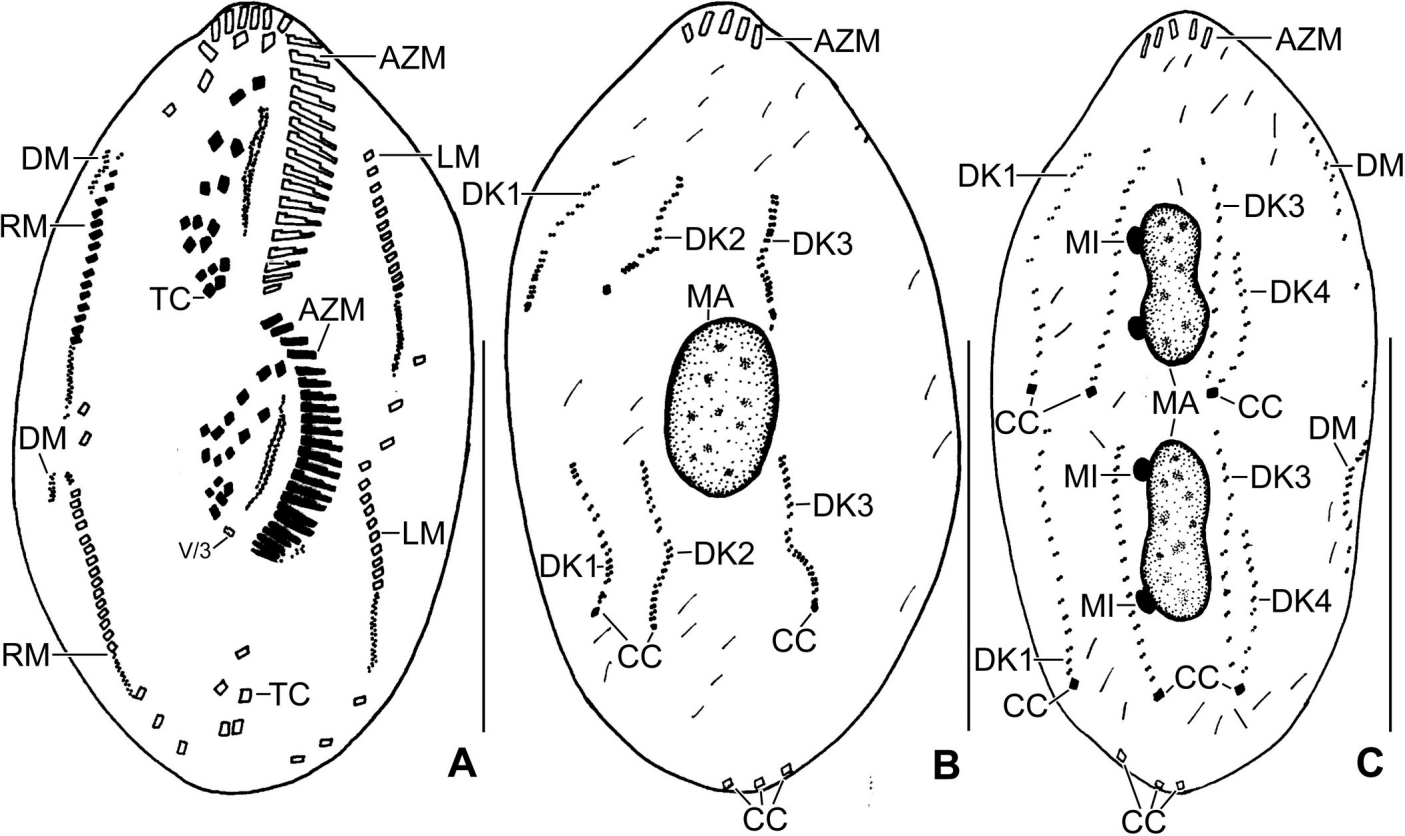
**Line diagrams of protargol stained middle (A, B) and late (C) dividers of *Sterkiella tricirrata* Italian population.** (A) Ventral surface (A), showing the formation of 16 fronto-ventral-transverse cirri from six anlagen (1:2:2:3:4:4). The newly formed fronto-ventral-transverse cirri migrate to their specific sites and dorsomarginal kineties develop close to the newly formed right marginal row. (B) Dorsal surface, showing the within row formation of dorsal kineties 1–3 at two levels. (C) Dorsal kinety 4 for the proter and the opisthe is formed by the simple fragmentation of dorsal kinety 3. AZM, adoral zone of membranelles; CC, caudal cirri; DK1–4, dorsal kineties; DM, dorsomarginal kinety; LM, left marginal row; RM, right marginal row; TC, transverse cirri; V/3, postoral ventral cirrus. Scale bars = 30 μm (A, B) and 40 μm (C).

**Fig 7:**
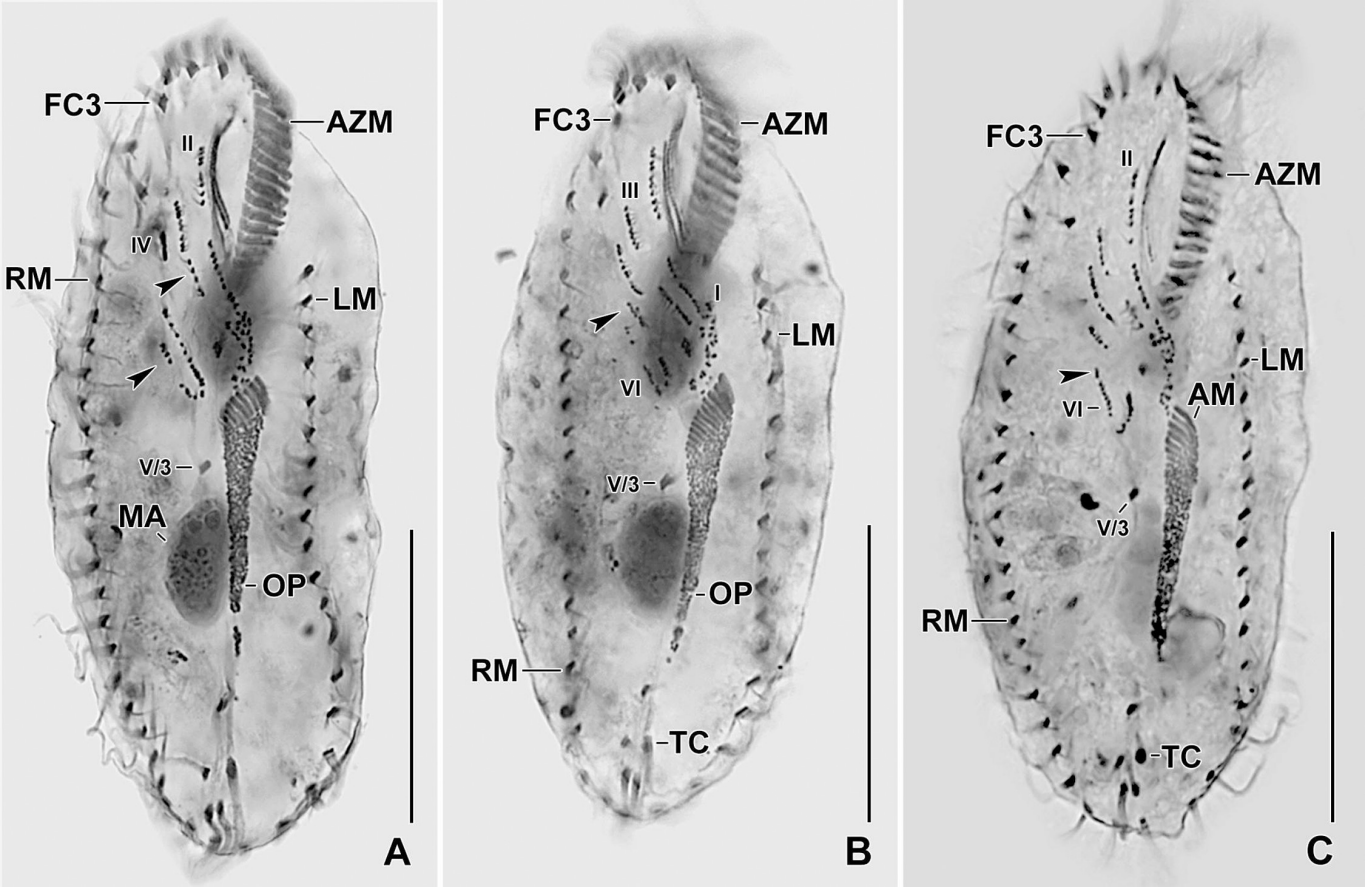
Photomicrographs of Protargol stained early dividers of *Sterkiella tricirrata* Italian population. **(A–C)** Anlage II of the opisthe moves anteriorly and merges with the anlage II of the proter. Arrowhead in (A) points to the part of the opisthe anlage III (note the orientation of the basal bodies and cilia) which probably joins the anlage III of the proter. Anlagen V and VI of the proter (arrowheads in B, C) originate from the anterior portion of the anlagen V and VI of the opisthe. AM, adoral membranelles; AZM, adoral zone of membranelles; FC3, frontal cirrus 3; LM, left marginal row; MA, macronuclear nodules; OP, oral primordium; RM, right marginal row; TC, transverse cirri; V/3, postoral ventral cirrus. Numerals denote cirral anlagen. Scale bars = 30 μm (E, F) and 40 μm (A–D, G).

**Fig 8:**
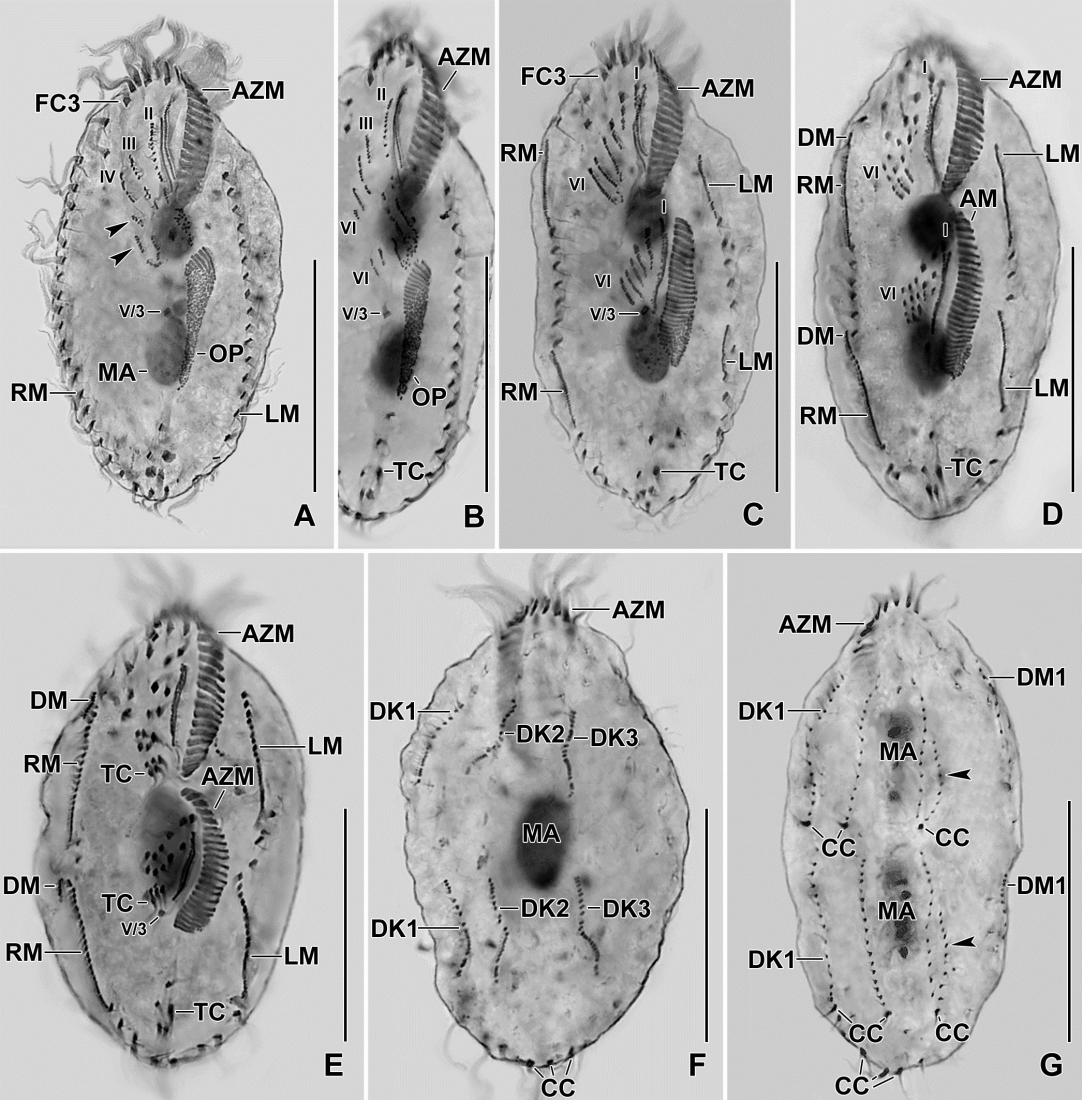
**Photomicrographs of protargol stained early (A–D), middle (E, F), and late (G) dividers of *Sterkiella tricirrata* Italian population.** For explanation refer to the legend of Fig 5B–5E. **(A)** Cirrus V/4 disaggregates and forms two anlagen for the opisthe, i.e., Anlagen V and VI; anterior portion of these anlagen proliferates anteriorly forming anlagen V and VI of the proter (arrowheads). **(B)** Cirrus IV/3 disaggregates and forms anlage IV of the proter. Cirrus IV/2 forms the anlage IV of the opisthe. **(C)** Six anlagen are formed both for the proter and the opisthe. Four anlagen for marginal cirri develop incorporating four to five parental marginal cirri in the proter and the opisthe. **(D, E)** The newly formed fronto-ventral-transverse cirri migrate to their specific sites and dorsomarginal kineties develop close to the newly formed right marginal row. **(F, G)** Within row formation of the anlagen for dorsal kineties 1–3 (F) takes place on the dorsal surface. Dorsal kinety 3 undergoes simple fragmentation forming kineties 3 and 4 (arrowheads in G). Caudal cirri are formed at the posterior end of dorsal kineties 1, 2, and 4, and the newly formed dorsomarginal kineties shift to the dorsal surface. AM, adoral membranelles; AZM, adoral zone of membranelles; CC, caudal cirri; DK1–3, dorsal kineties; DM1, dorsomarginal kineties; FC3, frontal cirrus 3; LM, left marginal row; MA, macronuclear nodules; OP, oral primordium; RM, right marginal row; TC, transverse cirri; V/3, postoral ventral cirrus. Numerals denote cirral anlagen. Scale bars = 30 μm (E, F) and 40 μm (A–D, G).

**Fig 9:**
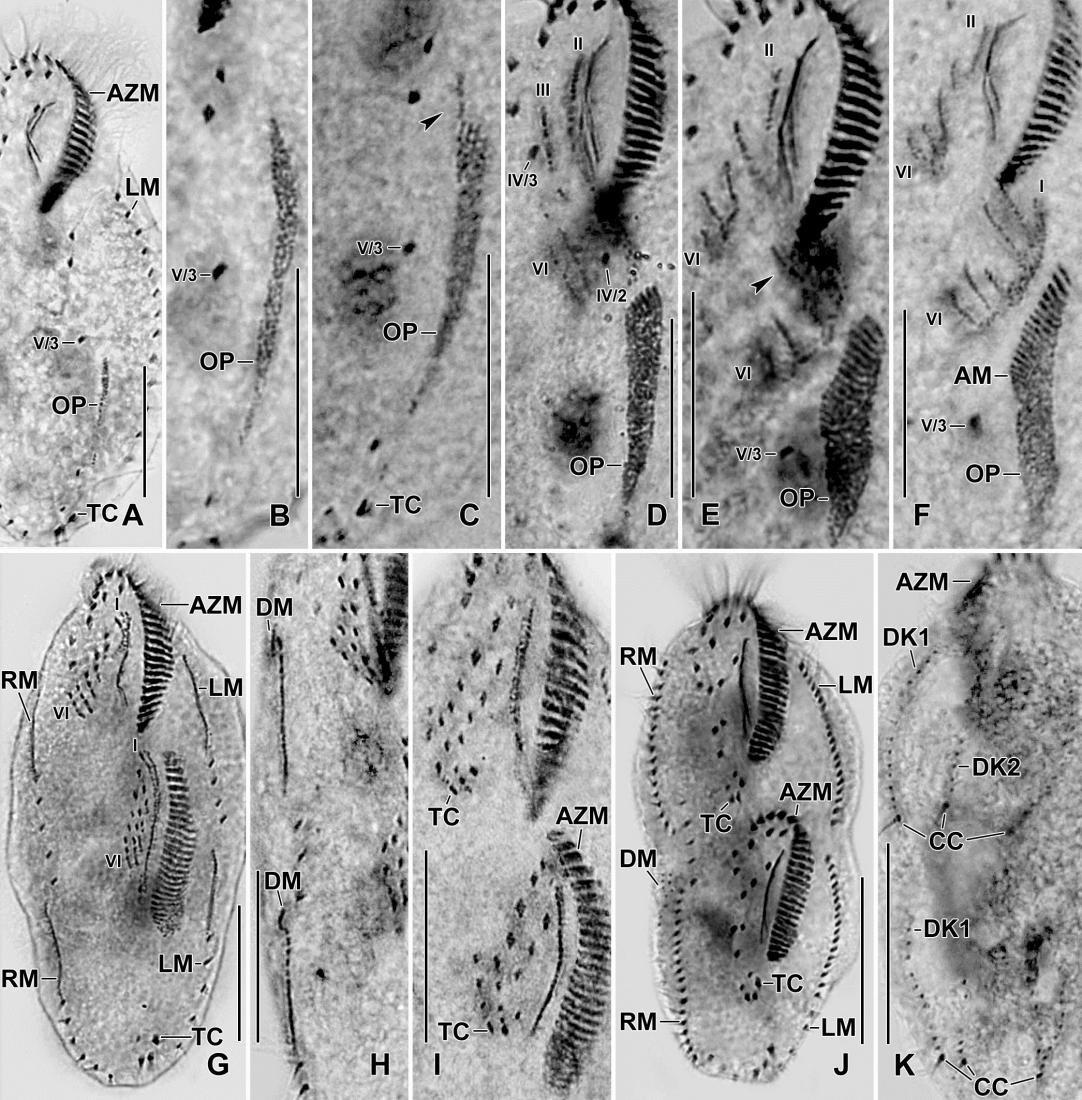
**Photomicrographs of protargol stained early (A–D), middle (E, F), and late (G) dividers of *Sterkiella tricirrata* Indian population.** The ontogenesis of Indian population is rather similar to Italian population except that a W-shaped pattern is formed for anlagen IV, V, and VI for the proter in the former (E, F). For explanation refer to the legend of Figs 5 and 8. Arrowhead in (E) points to anlage III of the opisthe. Six anlagen are formed both for the proter and the opisthe which splits transversely in 1:2:2:3:4:4 pattern. Dorsal kinety 3 undergoes simple fragmentation forming kineties 3 and 4; caudal cirri are formed at the posterior end of dorsal kineties 1, 2, and 4. AM, adoral membranelles; AZM, adoral zone of membranelles; CC, caudal cirri; DK1,2, dorsal kineties; LM, left marginal row; OP, oral primordium; RM, right marginal row; TC, transverse cirri; IV/3, fronto-ventral cirrus; V/3, IV/2, postoral ventral cirri. Numerals denote cirral anlagen. Scale bars = 20 μm (A–I) and 30 μm (J, K).

The ontogenetic stages of Italian and Indian population show a common origin of anlagen II, III, V, and VI for the proter and the opisthe (Figs 2H, 5A–5E, 6A–6C, 7A–7C, 8A–8G and 9A–9K). Difference in the anlagen formation was observed in the Indian population, i.e., a W-shaped formation for the anlagen IV, V, and VI of the proter during the late-early stage, similar to type species of the genus *Sterkiella* [18, 19] (Fig 9E and 9F).

The oral primordium originates close to transverse cirri IV/1 and extends towards the buccal vertex (Figs 2H, 5A, 7A–7C, 8A and 9A–9C). The scattered basal bodies at the anterior end of the oral primordium develop into the opistheʼs anlagen I–IV (Figs 5A, 5B, 7A–7C, 8A, 8B and 9A–9C). It is not clear whether disaggregating kinetosomes of cirrus IV/2 form or contribute to the formation of opistheʼs anlage IV (Figs 5B, 5C, 7A–7C, 8B, 8C and 9D–9F). Cirrus V/4 disaggregates and forms anlagen V and VI for the opisthe; anterior portions of the opistheʼs anlagen V and VI proliferate anteriorly, forming the proter’s anlagen V and VI (Figs 5A–5C, 7A–7C, 8A, 8B and 9D). Cirrus V/3 does not participate in anlagen formation (Figs 2H, 5A–5E, 6A, 7A–7C, 8A–8E and 9A–9F). Anlage II of the opisthe extends anteriorly crossing the buccal vertex and joining the disaggregating buccal cirrus in early dividers (Figs 5B, 5C, 7A–7C, 8A, 8B and 9D). Anlage I of the proter, i.e., the partially reorganized paroral and endoral, generates first frontal cirrus I/1 as well as the paroral and the endoral for the proter (Figs 5D, 5E, 6A, 8C–8E and 9F–9I). Cirri III/2 and IV/3 disaggregate and give rise to the anlagen III and IV of the proter (Figs 5B, 7A–7C, 8A, 8B, 9D and 9E). In the opisthe, anlage I separates from the posterior ends of anlagen II to IV and forms the paroral, endoral and cirrus I/1 (Figs 5D, 5E, 8C–8E and 9G–9I). Overall, five parental cirri and parental undulating membranes are involved in anlagen formation. The 16 frontal–ventral–transverse cirri arise from these anlagen, splitting in a 1, 2, 2, 3, 4, 4 pattern (Figs 5E, 6A, 8D, 8E and 9G–9J). No transverse cirri are formed from anlagen II and III. A new adoral zone of membranelles for the opisthe develops from the oral primordium, while the parental adoral zone of membranelles is retained unchanged for the proter.

The marginal anlagen arise at each of two levels by “within-row” anlagen formation utilizing one or two of the parental cirri at each level. The marginal anlagen elongate deploying four or five parental cirri and differentiate into new marginal rows. The remaining parental marginal cirri are resorbed (Figs 5D, 5E, 6A, 8C–8E, 8G, 8H and 8J).

On the dorsal surface, three anlagen are formed within row from dorsal kineties 1, 2 and 3 at two levels, (one set for the proter and one for the opisthe) (Figs 6B, 6C, 8F, 8G and 9K). The third dorsal primordium fragments at the middle giving rise to the third and fourth kineties. The two dorso-marginal rows arise close to the anterior of right marginal row anlagen (Figs 6B, 6C, 8F, 8G and 9K). Caudal cirri originate at the posterior end of the newly formed dorsal kineties 1, 2, and 4 (Figs 6C, 8G and 9K).

Nuclear division proceeds in the usual manner, i.e., in mid-dividers the macronuclear nodules fuse to form a single mass which divides twice to produce the typical four nodules in late dividers (Figs 6B, 6C and 8C–8G). The micronuclei undergo mitotic division.

### SSU rRNA gene sequence and phylogeny

**Fig 10:**
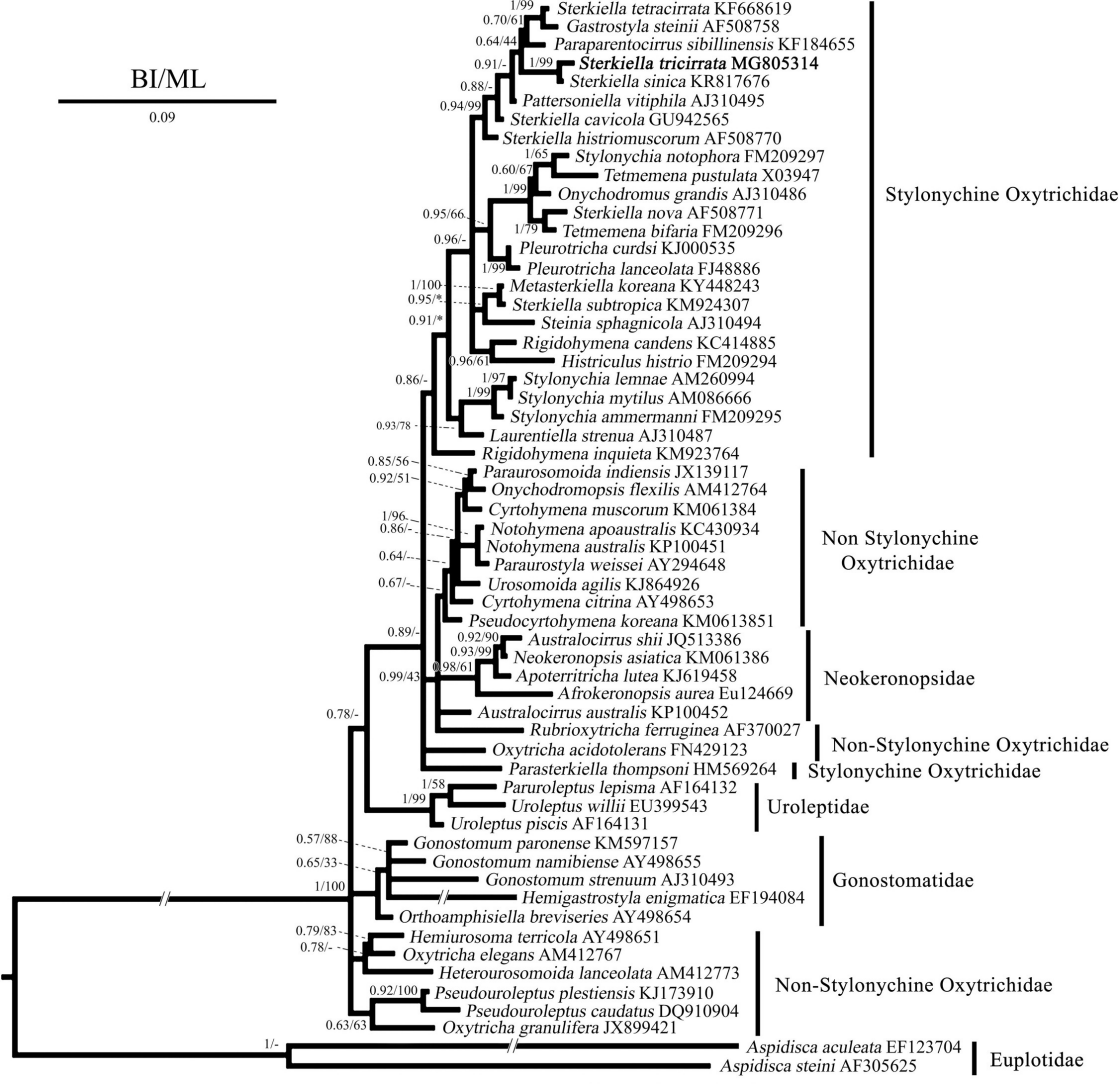
Bayesian tree inferred from the SSU rRNA gene sequences, showing the phylogenetic position of *Sterkiella tricirrata* (bold). Codes following the names are GenBank accession numbers. Numbers at the nodes represent the Bayesian inference (BI) posterior probabilities and the maximum likelihood bootstrap values out of 1000 replicates. A hyphen (-) represents differences between the BI and ML tree topologies. Asterisks represents values lower than 40%. The scale bar corresponds to nine substitutions per 100 nucleotide positions.

The SSU rRNA gene sequence of *Sterkiella tricirrata* Italian population is 1,628 bp in length and has a GC content of 45.15%. It has been deposited in the NCBI database under the accession number MG805314. Phylogenetic trees inferred from the SSU rRNA gene sequences using ML and BI present similar topologies; thus, only the BI tree is shown here (Fig 10). *Sterkiella tricirrata* clusters with *Sterkiella sinica* (1.00 BI and 99% ML) within the stylonychine oxytrichids group.

## Discussion

### Comparison of *Sterkiella tricirrata* with related species and populations

*Sterkiella tricirrata* can be compared with species of the genus *Sterkiella* having two macronuclear nodules, i.e., *Sterkiella histriomuscorum* (Foissner et al., 1991) Foissner, Blatterer, Berger & Kohmann, 1991; *S*. *nova* Foissner & Berger, 1999; *S*. *subtropica* Chen et al., 2015; *S*. *sinica* Chen et al., 2016, and *S*. *ecuadoriana* Foissner & Heber in Foissner, 2016. *Sterkiella tricirrata* mainly differs from all the above mentioned species in having invariably three (vs. four or five) transverse cirri. Apart from transverse cirri, the Italian and Indian populations of *S*. *tricirrata* possess lower number of adoral membranelles (20–26 and 21–26 vs. 26–44 average from populations described) in comparison with *S*. *histriomuscorum* [11]. There is one population of *Sterkiella histriomuscorum* with four transverse cirri [18]; however, a reinvestigation on the number of transverse cirri and the ontogenetic pattern is needed for a better comparison. The invariably three transverse cirri (over 250 specimens analyzed from Italian population and 50 specimens from Indian population) is a constant character of *Sterkiella tricirrata*. This, supports the validity of *Sterkiella tricirrata* at species rank and out of the *S*. *histriomuscorum* complex; this separation is in agreement with Kumar et al. [1], who described a novel *Sterkiella* species with four macronuclear nodules mainly on the basis of four transverse cirri. *Sterkiella histriomuscorum* and *S*. *nova* are indistinguishable based on the morphology and gene sequence data are required for their identification [18]. *Sterkiella subtropica* can be separated from *S*. *tricirrata* Italian and Indian populations by the marine vs. terrestrial habitat, large body size in vivo 100–200 × 35–70 μm (vs. 70–90 × 30–50 μm), slightly higher number of adoral membranelles 25–39 (vs. 20–26 and 21–26), and number of right 19–27 (vs. 18–21 and 14–18) and left 18–26 (vs. 14–18 and 13–16) marginal cirri [16]. The Italian and Indian populations of *Sterkiella tricirrata* can be separated from *S*. *ecuadoriana* by the slightly larger body length (63–86 μm and 61–74 vs. 91–150 μm), adoral membranelles (20–26 and 21–26 vs. 32–49) and bristles in DK1 (13–18 and 17–22 vs. 27–45) [3]. *Sterkiella sinica* can be distinguished from Italian and Indian populations of *S*. *tricirrata* in having an extra cirrus between the second and third frontal cirri (vs. no such cirrus), distance between cirri V/2 and V/3 24.5 μm (vs. 19.3 and 10.6 μm) and cirri V/3 and V/4 4.7% (vs. 9.4% and 16.1%) of the body length, number of cirri in left 19–23 (vs. 14–18 and 13–16) marginal row, and by the lower number of bristles in DK1 (22–25 vs. 13–18 and 17–22), DK2 (20–24 vs. 14–21 and 14–20), DK3 (14–19 vs. 10–14 and 7–14), DK4 (13–19 vs. 8–13 and 11–15), DM1 (9–15 vs. 5–8 and 7–10), and DM2 (5–10 vs. 2–5 and 3–6) [31]. Minor differences between *Sterkiella sinica* and Italian and Indian populations of *S*. *tricirrata* were observed in the body size 85–110 × 35–45 μm (vs. 70–90 × 30–50 μm) and number of cirri in right 18–22 (vs. 18–21 and 14–18) marginal row [31].

Other than species of the genus *Sterkiella*, *Sterkiella tricirrata* can be compared with *Parasterkiella thompsoni* (Foissner, 1996) Küppers et al., 2011; *Metasterkiella koreana* Kumar et al., 2017; and *Fragmospina depressa* Foissner, 2016. *Sterkiella tricirrata* mainly differs from *Parasterkiella thompsoni* in having two (vs. three) macronuclear nodules, numbers of dorsal kineties 6 (vs. 5), and presence (vs. absence) of fragmentation of dorsal kinety 3 during dorsal morphogenesis [17]. It differs from *Metasterkiella koreana* mainly in the number of transverse cirri (invariably 3 vs. 5) and in the ontogenesis, i.e., cirrus V/3 intact (vs. involved during anlagen formation) [2]. *Fragmospina depressa* can be separated from *S*. *tricirrata* by having a paroral membrane close (vs. distant) to the adoral membranelles, number of transverse cirri 5 (vs. invariably 3) and the structure of resting cyst, i.e., spinous (vs. wrinkled) surface [3].

The Indian population of *Sterkiella tricirrata* shows minor differences in size and ciliature with the Italian population as mentioned in the description section. The resting cyst of Indian population appears to be smooth (vs. wrinkled in Italian population); however, additional data on the resting cyst of the Indian population is required to confirm this feature. The original population described by Buitkamp [32] could not be meaningfully compared since most of the morphometric data are lacking. Main differences observed (data from the single image of a protargol stained specimen provided in Buitkamp [32] rely in the number of cirri in left (10 vs. 16 and 20 in Indian and Italian populations, respectively) and right (12 vs. 14 and 16 in Indian and Italian populations, respectively) marginal rows. Further, the original description of *S*. *tricirrata* mentioned the presence of five (instead of six recorded in the present study) dorsal kineties. We agree with Berger [11] since the dorsal kinety 6 is rather short it could have been easily missed by Buitkamp [32]. A reinvestigation of the Ivory Coast population will further clarify if it requires separation at the species/subspecies level.

### Notes on the ontogenesis of the genus *Sterkiella*

Berger and Foissner [15] reported that the anlagen V and VI of the opisthe originate de novo in species of the genus *Sterkiella*. Later, Foissner et al. [19] provided a detailed ontogenetic data on *Sterkiella cavicola* (Kahl, 1935) Foissner, Blatterer, Berger & Kohmann, 1991, correcting the previous observations that the anlagen V and VI originate by disaggregation of the cirrus V/4. The same pattern is observed also for *Sterkiella tricirrata* where cirrus V/4 generates anlagen V and VI of the opisthe; however the anterior patches of both the anlagen move anteriorly and later form the proter anlagen V and VI (Table 2). On the contrary, anlagen V and VI of the proter originate from a disaggregation of cirrus IV/3 in *S*. *cavicola* [19]. Further, the ontogenetic data of *S*. *tricirrata* shows that the anterior portions of anlagen II and III of the opisthe proliferate anterior and merge with the disaggregating cirri II/2 and III/2 respectively to form anlagen II and III of the proter. Recently, Kumar et al. [2] erected a novel genus, *Metasterkiella*, for a species having similar morphological features as that of *Sterkiella histriomuscorum*; however, the former not only showed difference in the anlagen formation but also the involvement of cirrus V/3 in anlagen formation, a feature never reported for any stylonychid ciliate. Possibly the involvement of cirrus V/3 during anlagen formation and the semi-rigid body indicate that the *M*. *koreana* might have recently evolved from an *Oxytricha*-like ancestor. As mentioned above, *Sterkiella tricirrata* also shows differences with *Sterkiella cavicola* in the formation of anlagen II, V and VI, i.e., confluent anlagen II and anlagen V and VI of the opisthe give rise to anlagen V and VI of the proter by enlargement and then splitting, though cirrus V/3 remains intact during ontogenesis. The ontogenetic difference between the Indian and Italian populations, i.e., formation of a W-shaped pattern (vs. separate) by the anlagen IV, V, and VI of the proter in late-early divider, indicates that the Indian population may represent a separate subspecies/species if the pattern mentioned is found to be stable in other populations with consensus of molecular data. As of now, we do not perform its separation from the *Sterkiella histriomuscorum* complex and wait for further data to resolve the phylogenetic status of the species within the complex. However, the different morphogenetic patterns, within the genus *Sterkiella*, as seen in the present study and Foissner et al. [19] needs to be reflected in the generic characteristics, thus we have provided an improved diagnosis of the genus *Sterkiella*. The Austrian population of *Sterkiella histriomuscorum* shows some similarity in anlagen formation with *S*. *tricirrata* [18, 33]; however, a detailed investigation of its morphogenesis is required for a reliable comparison.

**Table 2:** Comparison between species of the genus *Sterkiella*.

Characteristic	*Sterkiella tricirrata*	*Sterkiella histriomuscorum*	*Sterkiella histriomuscorum*	*Sterkiella nova*	*Sterkiella cavicola*
	Italy	Austria	Antarctica	USA	Austria
Early disaggregation of cirrus II/2 during ontogenesis	Present	Present	Absent	Absent	Absent
W-shaped anlagen formation	Absent	Absent	Present	Present	Present
Formation of confluent anlagen II for the proter and opisthe	Present	Present	Absent	Absent	Absent
Formation of anlagen V and VI of the proter from anlagen V and VI of the opisthe	Present	Present	Absent	Absent	Absent
Transverse cirri, number	3	4	5	5	5
Data source	Present study	Berger et al. [34]	Petz & Foissner [33]	Foissner & Berger [18]	Foissner et al. [19]

### Phylogenetic position of *Sterkiella tricirrata*

*Sterkiella tricirrata* clusters with *S*. *sinica* (1.00 BI and 99% ML; Fig 9) within the stylonychine oxytrichids, in a clade away from the type species (*Sterkiella cavicola*) of the genus *Sterkiella*; we assume that the molecular relatedness of *S*. *tricirrata* and *S*. *sinica* could be because of similarity in the formation of anlagen. However, a detailed investigation of the ontogenesis of *S*. *sinica* is needed to properly compare these genetically similar species. Our phylogenetic analyses also shows that *S*. *histriomuscorum* and *S*. *cavicola* behave as sisters of a larger clade containing, in addition to the aforementioned *S*. *tricirrata* + *S*. *sinica* sub-clade, a further high supported sub-clade including *S*. *tetracirrata* + *Gastrostyla steinii* (1.00 BI, 99% ML). The other *Sterkiella* species are distributed across two more clades: i) *S*. *nova* with *Tetmemena bifaria* (1.00 BI, 79% ML); and ii) *S*. *subtropica* with *Metasterkiella koreana* (1.00 BI, 100% ML). The monophyly of the genus *Sterkiella* is not supported in our phylogenetic analyses as also evident in other recent studies [1, 2, 15–17]. Certainly, more sequences from populations of the *S*. *histriomuscorum* complex as well as from other *Sterkiella* species are required to obtain better resolution. Although in recent years, the situation has been slightly improved with the establishment of genera, namely, *Parasterkiella* Küppers et al., 2011 and *Metasterkiella* Kumar et al., 2017. *Sterkiella subtropica* and *S*. *nova* cluster away from the type species (*S*. *cavicola*); for the former species, Kumar et al. [2] suggested that it probably belongs to the genus *Metasterkiella* due to highly similar morphology and gene sequence; this interpretation is also supported by our phylogenetic analyses. The classification of *Sterkiella nova* has been widely debated among classical taxonomists and molecular biologists who established this species as model organism for analyzing various biological phenomena such as epigenetic inheritance, genome rearrangement, somatic differentiation and many others [11, 18]. In this regards, Foissner and Berger [18] described *S*. *histriomuscorum* and *S*. *nova* in great detail from viable genetic systems (*via* frozen resting cysts) established by molecular biologists. They mentioned that both species are inseparable based on the morphological characters; though based on the differences in molecular sequences of actin I and DNA pol α genes, they proposed them as different species. Considering the complexity of identification it is unclear whether the gene sequences provided by Hewitt et al. [35], which is used in the present study and remains the only sequence available for *S*. *nova*, is of same species described by Foissner and Berger [18].

As of now, only differences which seem most suitable to solve the polyphyletic behavior of the genus *Sterkiella* is the data on the ontogenetic pattern on the ventral and dorsal surface. In *Sterkiella cavicola* anlagen V and VI of the proter originate from cirrus IV/3 forming W-shaped anlagen [19], whereas it forms from opistheʼs anlagen V and VI during the early ontogenetic stages in *Sterkiella tricirrata* and the genus *Metasterkiella*. In our phylogenetic tree, *Metasterkiella* forms a distant clade away from that of *Sterkiella tricirrata* the involvement of cirrus V/3 (vs. intact) during anlagen formation possibly justifies this distant relationships. Nonetheless, several examples exists like, *Parasterkiella thompsoni*, *Fragmospina depressa*, which would have been easily identified as *Sterkiella* species but separated based on detailed investigations on morphology and cyst structure. *Parasterkiella thompsoni* shows a different ontogenetic pattern on the dorsal surface and acquires a place distant from *Sterkiella* species [17], for the species of the genus *Fragmospina* no gene sequence is available thus far. We believe that addition of related molecular sequences, e.g., *Fragmospina*, *S*. *histriomuscorum* populations, and gene sequences from other loci will further support the monophyly of the genus *Sterkiella*.

### Soil ciliate diversity and species identification: A contribution

Ciliated protists are a highly diverse group of microbial eukaryotes that play a key role in soil microbial food webs by mediating the fluxes of nutrients and energy between different trophic levels [36]. Nevertheless, ciliate diversity in the soil is a still largely neglected research topic and this taxon is significantly less studied than other soil microbial taxa such as bacteria and fungi [37, 38]. Since 2009, our group has made a significant contribution to in-depth knowledge about the diversity of soil ciliates across two continents, i.e., Europe (Italy) and Asia (India and South Korea) [1, 9, 39–45]. Numerous faunistic surveys performed in the framework of several projects, allowed us to isolate and describe several novel species and genera, as well as re-describe poorly known or even misidentified species [9, 39–45]. According to Foissner [46], more than 70–80% of the soil ciliate diversity is still unexplored and a single soil sample can host new species/genera such as in the case of the soil sample collected from the regional Park of Colfiorito described in this study, in which one new and one poorly known species were identified, i.e., *Pseudouroleptus plestiensis* [45] and *Sterkiella tricirrata* (present study).

In the end, our sampling effort has allowed us to contribute to strengthen the knowledge about soil ciliate diversity, providing hints about their biogeographic distributions and new distinguishing characters (i.e., cyst morphology, molecular data, ontogenetic processes, arrangement and number of cirri, etc.) among hypotrich ciliates that can be helpful in species identification within problematic (cryptic) species "complexes".

**Phylum Ciliophora Doflein**, **1901**
**Class Spirotrichea Bütschli**, **1889**
**Order Sporadotrichida Fauré-Fremiet**, **1961**
**Family Oxytrichidae Ehrenberg**, **1838**

## Genus *Sterkiella*

### Improved diagnosis

Body semi-rigid. Eighteen or less frontal-ventral-transverse cirri arranged in typical oxytrichid pattern. One right and one left row of marginal cirri. Six dorsal kineties including dorsomarginal rows, kinety 3 with simple fragmentation; caudal cirri present. Undulating membranes in *Oxytricha* pattern. Opisthe's anlage II may contribute to proter's anlage II. Anlagen V and VI of the proter originate from cirrus IV/3 forming W-shaped anlagen or from anlagen V and VI of the opisthe.

## Sterkiella tricirrata

### Improved diagnosis (averages are from the populations of India, Italy, and Ivory Coast)

Size about 80 × 40 μm in vivo; body elongate to broadly ellipsoidal. Nuclear apparatus composed of two macronuclear nodules and two micronuclei on average. Invariably, 16 frontal-ventral-transverse cirri, including three transverse cirri. Right and left marginal rows composed of an average of 15 and 14 cirri, respectively. Adoral zone 37% of body length and composed of an average of 23 membranelles. Three narrowly spaced, inconspicuous caudal cirri. Resting cyst with wrinkled surface. Soil habitat.

### Neotype material

Since the original description is incomplete and no type material is available thus according to the Article 75.3 of the ICZN (1999) we propose neotypification of the *Sterkiella tricirrata* with sampling site of the Italian population being the type locality. Two neotype slides of Italian population containing the protargol stained neotype specimen (Figs 2F and 3A) and relevant morphostatic specimens have been deposited at the Natural History Museum, London, UK, with registration numbers NHMUK 2014.3.20.1 and NHMUK 2014.3.20.2. Further, two slides of the Indian population are deposited at the Natural History Museum, London, UK, with registration numbers NHMUK 2011.7.4.2 and NHMUK 2011.7.4.3 and one at the type collection of the Zoological Survey of India, Kolkata, India, with registration number Pt 3067. The SSU rRNA gene sequence is deposited in GenBank (accession number: MG805314).

### Occurrence and ecology

Buitkamp [32] isolated *Sterkiella tricirrata* from the soil collected from the burnt savannah in the Ivory Coast. The Italian population was identified from the ‘Molinaccio’ site during the summer (dry season), where it was moderately abundant in non-flooded Petri dish culture. For details on the soil physico-chemical parameters and other ciliate species identified in the same soil sample, refer to Bharti et al. [45]. The Indian population was identified from the soil sample collected from the tracts of the tropical rain forest of the Silent Valley National Park, India. For details on other ciliates species identified from the soil samples collected, refer to Kumar et al. [47]. Feeds on bacteria, small amoeba, and flagellates; clonal cultures can be raised as mentioned in materials and methods section.
